# Muscle Strength Estimation of Key Muscle–Tendon Units During Human Motion Using ICA-Enhanced sEMG Signals and BP Neural Network Modeling

**DOI:** 10.3390/s25206273

**Published:** 2025-10-10

**Authors:** Hongyan Liu, Jongchul Park, Junghee Lee, Dandan Wang

**Affiliations:** Department of Marine Convergence Design Engineering, Pukyong National University, 45, Yongso-ro, Nam-gu, Busan 48513, Republic of Korea; liuhongyan@pukyong.ac.kr (H.L.); ljh@pknu.ac.kr (J.L.); wdd703@pukyong.ac.kr (D.W.)

**Keywords:** independent component analysis algorithm, muscle–tendon unit, BP, muscle strength prediction, PCC

## Abstract

Accurately predicting the muscle strength of key muscle–tendon units during human motion is vital for understanding movement mechanisms, optimizing exercise training, evaluating rehabilitation progress, and advancing prosthetic control technologies. Traditional prediction methods often suffer from low accuracy and high computational complexity. To address these challenges, this study employs independent component analysis (ICA) to predict the muscle strength of tendon units in primary moving parts of the human body. The proposed method had the highest accuracy in localization, at 98% when the sample size was 20. When the sample size was 100, the proposed method had the shortest localization time, with a localization time of 0.025 s. The accuracy of muscle strength prediction based on backpropagation neural network for key muscle–tendon units in human motion was the highest, with an accuracy of 99% when the sample size was 100. The method can effectively optimize the accuracy and efficiency of muscle strength prediction for key muscle–tendon units in human motion and reduce computational complexity.

## 1. Introduction

Sports biomechanics is an interdisciplinary field that explores the influence of external forces (such as gravity and air resistance) and internal forces (such as muscle contraction) on human movement [1]. It is of great significance in rehabilitation training, improving athletic performance, and prosthetic control. Skeletal muscle, as the key actuator driving movement, is composed of multiple muscle–tendon units (MTUs). The coordinated activation patterns of each MTU in different movements determine the motor function [2,3]. Therefore, accurately identifying the key MTU and its muscle strength status is the foundation for achieving personalized training and auxiliary control. Surface electromyography (sEMG), as a non-invasive and easy-to-operate method for collecting bioelectric signals, can reflect muscle activity status in real time and has been widely used in human motion analysis [4,5]. However, sEMG signals usually have high dimensions, strong noise, and are vulnerable to muscle cross-talk interference, making it difficult for traditional MTUs muscle strength modeling methods to accurately capture the nonlinear and dynamic characteristics of muscle activities, and the predictive ability is limited [6]. Independent component analysis (ICA) is a feature extraction method commonly used for signal source separation, which can separate statistically independent source components from observed signals [7,8]. ICA does not require prior knowledge and has the potential to extract target muscle activation components from mixed sEMG signals, which is expected to improve the localization accuracy of key MTUs and provide a clear input basis for muscle strength prediction.

ICA has achieved remarkable results in areas such as heart rate estimation, electroencephalogram (EEG) noise reduction, load identification, and vital sign detection, demonstrating its advantages in complex physiological signal processing. For example, Gupta et al. proposed an ICA-based method that was robust to motion and lighting artifacts, achieving minimal estimation error in heart rate prediction [9]. Agarwal et al. combined sliding singular spectrum analysis with ICA for EEG denoising in alcohol detection, reaching a 98.97% classification accuracy with reduced computational complexity [10]. Although EEG and sEMG have different signal sources, both belong to high-dimensional non-stationary physiological electrical signals and face signal aliasing and noise interference. Therefore, the successful application of ICA in EEG provides theoretical and methodological support for its feasibility in sEMG signal separation. Wang et al. improved component identification using a semidefinite programming algorithm based on Lagrangian first-order information [11]. In power systems, Zhang et al. used a modified FastICA to isolate harmonic loads under noisy conditions [12]. Qi et al. validated the derivative ICA for non-contact estimation of respiration and heart rate via radar [13].

Muscle strength prediction is critical in rehabilitation, sports training, and biomechanics. It can quantify muscle function, support training optimization, prosthetic control, and injury prevention [14]. Various strategies have been proposed to enhance muscle modeling. Zaman et al. developed a hybrid model combining motion prediction and muscle dynamics using a 2D skeletal system [15]. Sharma et al. introduced a machine learning strategy to reduce reliance on complex upper-limb models, achieving a 0.23 error rate [16]. O. Keeffe et al. proposed a non-parametric framework for strength estimation based on sEMG, which could effectively monitor coordinated changes related to fatigue [17]. Zhou et al. improved ankle joint torque prediction under various postures with non-negative matrix factorization, with an accuracy rate of 94.15% [18]. Bennett et al. enhanced knee joint strength prediction using static optimization and randomized sEMG input, achieving errors ranging from 192 to 674 N and capturing 98% of measured loads during standing [19].

In summary, although ICA and neural networks have shown great potential in signal processing and modeling, their comprehensive application in predicting key MTUs during human motion is still relatively insufficient. To improve accuracy and real-time performance, this study proposes a novel method combining ICA-enhanced sEMG signal separation with backpropagation (BP) neural network modeling. The proposed method aims to accurately locate key MTUs and estimate muscle strength. The innovation of this study lies in applying ICA for the first time to localize key MTUs in human motion, enabling deeper insight into the functional roles of different muscle groups during specific actions and enhancing strength prediction accuracy. The proposed framework helps to better understand muscle function and promote the development of biomechanics by reducing signal mixing and improving analysis accuracy.

## 2. Methods for Predicting Muscle Strength of Key Muscle–Tendon Units in Human Movement

### 2.1. Key MTU Localization of Human Motion Based on Independent Component Analysis Algorithm

Before predicting the muscle strength of the key MTUs in human motion, this study first located the MTUs. The reason is that the exact location and structure of MTUs directly affect the generation and transmission of muscle strength. As the connecting part between muscles and bones, the integrity and accurate spatial position of tendons determine how muscle strength is effectively converted into body motion [20,21]. By accurately locating MTUs, the mechanism of action of different muscle groups in specific movements is obtained, thereby making muscle strength prediction more precise. In addition, correct positioning can help identify possible pathological changes in MTUs, which is crucial for designing effective rehabilitation plans and optimizing exercise performance. Therefore, MTU localization research not only provides necessary basic information for muscle strength prediction, but also serves as a prerequisite for ensuring prediction accuracy and practicality.

Traditional preprocessing methods for sEMG signals, such as band-pass filtering, wavelet denoising, or mean normalization, are primarily designed to remove high-frequency noise and baseline drift. However, they are often insufficient when addressing signal mixing caused by the coordinated activity of multiple muscle groups. In high-dimensional sEMG data, electrical signals from different muscles tend to overlap significantly, which directly affects the accuracy of identifying target MTUs. To address this issue, this study introduces the ICA method, which decomposes multiple mixed signals into statistically independent components, thereby effectively separating source signals from different muscles in high-dimensional sEMG data. Compared with other dimensionality reduction or filtering techniques, ICA does not rely on the assumption of Gaussian distribution and offers stronger adaptability and robustness [22,23], making it particularly suitable for unsupervised multi-source signal separation tasks. Therefore, using ICA for the localization of key MTUs not only enhances the signal representation of target muscle groups but also provides a more reliable input foundation for subsequent muscle strength modeling.

**Figure 1:**
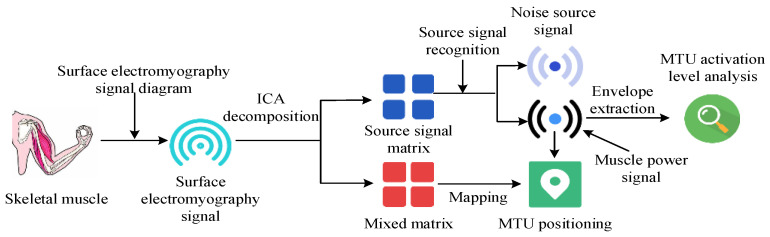
Diagram of the key MTU localization steps for human motion based on ICA.

The sEMG data were collected using disposable surface electrodes (Ambu Inc., Columbia, MD, USA) and amplified with a Trigno Wireless EMG System (Delsys Inc., Natick, MA, USA). Motion capture was performed using a Vicon system (Oxford Metrics, 6 Oxford Pioneer Park, Yarnton, Oxfordshire, UK, OX5 1QU), and muscle oxygenation was measured using Moxy monitors (Fortiori Design LLC, 1155 West Shore Dr SW, Hutchinson, MN, USA, 55350). Data analysis was conducted in MATLAB (R2022b, MathWorks, Natick, MA, USA) with the ICA algorithm implemented in Python (version 3.10, https://www.python.org/, accessed on 20 June 2024). The specific process is illustrated in Figure 1.

**Figure 2:**
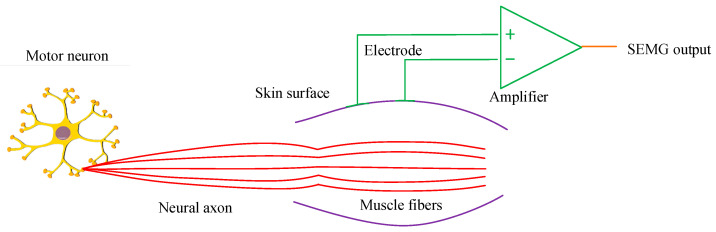
sEMG signal acquisition of target muscle area.

In Figure 1, the steps for locating the key MTUs of human motion based on ICA are as follows. First, the high-density sEMG signal is separated and the original signal and associated mixing matrix are extracted. Secondly, the matrix obtained from the above steps is analyzed using spectral analysis methods. By analyzing the spectral characteristics of each component, the true source of electromyography signals and the source of electromyography signals affected by factors such as noise are identified. Finally, based on the correspondence between the array electrodes and the electromyography signal source, the specific locations of MTUs are determined. When locating the key MTUs of human motion, the first step is to collect the electromyography signals of the target muscle area. sEMG signals are biological currents produced by contracted muscles. The nervous system controls muscle activity. Different signals are generated simultaneously by different muscle fiber motor units on the skin surface. sEMG is a safe, easy-to-use, non-invasive electromyography recording technique, which can objectively quantify muscle energy. It is a frequently used electromyography signal acquisition strategy [24,25]. Therefore, this study used sEMG to collect electromyography signals of the target muscle area, as shown in Figure 2.

As shown in Figure 2, sEMG was used to collect electromyography signals of the target muscle area. The electrode is placed on the surface of the human skin. This records the small potential difference caused by muscle contraction on the skin surface. Then, the electromyographic acquisition circuit is amplified and converted to form surface electromyographic signals that can be used for processing. sEMG signals are easily affected by environmental noise, electromyography noise, and power interference. Therefore, the collected sEMG signals are preprocessed to remove noise and improve signal quality. The steps for preprocessing sEMG signals using this method are as follows. First, Fourier transform is performed on the signal. Then, a Butterworth low-pass filter is used to filter the spectrum. Finally, inverse Fourier transform is performed on the filtered spectrum to obtain the denoised sound signal. Fourier transform is then used to change time-domain signals into frequency-domain signals.

When using ICA to separate high-density sEMG signals, it is assumed that there are multiple observation signals represented as $x\left(t\right)$, which are linearly mixed from several independent signal sources $s\left(t\right)$. $x\left(t\right)$ is shown in Equation (1).

(1) x(t)=As(t)+n(t)

**Figure 3:**
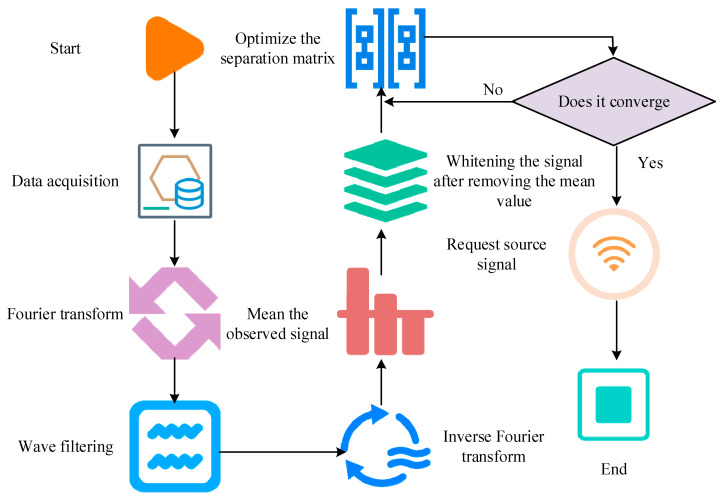
Step diagram of high-density sEMG signal separation based on ICA algorithm.

In Equation (1), A represents the mixed matrix. $n\left(t\right)$ represents the noise. ICA is used to obtain a separation matrix W, resulting in y(t)=Wx(t). $y\left(t\right)$ is an estimate of the independent signal source $s\left(t\right)$. The steps of using ICA to separate high-density sEMG signals are as follows. First, the observation signal is centered by subtracting the mean to eliminate the influence of the direct-current component. Further whitening transforms the covariance matrix of the observed signal into an identity matrix, removing the correlation of the signal. Selecting the separation matrix W is a crucial step. Its goal is to convert the whitened observation signal into an estimated independent signal source, typically using maximum likelihood estimation to find the appropriate W. Finally, the estimated separation matrix W is used to obtain the estimated independent signal source $s\left(t\right)$. The steps for separating high-density sEMG signals using ICA are shown in Figure 3.

The centralization is to remove the mean of data and make it zero. For the observation signal $x\left(t\right)$, the centralized signal $x^{*}\left(t\right)$ can be obtained by Equation (2).

(2) $$x^{*}\left(t\right)=x\left(t\right)-\mu$$

In Equation (2), μ represents the mean of $x\left(t\right)$. Whitening is the process of transforming a signal into a statistically independent signal with unit variance. The covariance matrix of $x^{*}\left(t\right)$ is B, and the whitening change Z is shown in Equation (3).

(3) $$Z=W_{Z}x^{*}\left(t\right)$$

In Equation (3), $W_{Z}$ represents the whitening matrix, which is usually composed of the inverse square root of the eigenvalue matrix of B. In maximum likelihood estimation, it is usually assumed that the probability density function of the source signal is known. The separation matrix W is estimated by maximizing the logarithmic likelihood function of the observed data. For a given observation signal $x\left(t\right)$, the likelihood function L is expressed as Equation (4).

(4) $$L=\sum_{t=1}^{T}\log p\left(x(t)\right)$$

In Equation (4), p signifies the joint probability density function given W. T is the number of observed data. The independent signal source $s\left(t\right)$ is shown in Equation (5).

(5) $$s\left(t\right)=Wx\left(t\right)$$

In Equation (5), W represents the separation matrix.

### 2.2. Muscle Strength Prediction and Evaluation of Key MTUs in Human Motion

**Figure 4:**
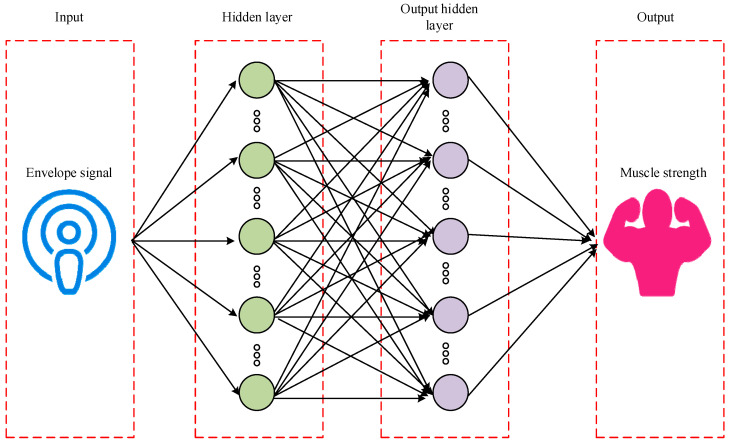
Muscle strength prediction of human motion key MTUs based on BP.

After locating the key MTUs of human motion based on the ICA algorithm, the muscle strength prediction and evaluation of human motion key MTUs was performed. Backpropagation (BP) is a multi-layer feedforward network trained by error, which is the most extensively applied method. BP adds one or more layers of neurons between the input layer and the output layer, namely hidden units. These are not directly connected to the outside, but changing their state can affect the ratio of input to output [26,27]. The advantage of this approach lies in its powerful nonlinear fitting ability, which can handle complex input and output relationships and can optimize its predictive performance by learning rich training data. In contrast, BP can better capture the complex mapping relationship between electromyography signals and muscle strength, thereby improving the prediction accuracy [28]. Therefore, the study employed a BP neural network to predict the muscle strength of key MTUs in human motion. Muscle strength prediction of key MTUs in human motion based on BP is displayed in Figure 4.

In Figure 4, the core step in predicting muscle strength of key MTUs based on BP is transmitting electromyography signals to the hidden layer nodes, and finally applying them to the output layer to achieve nonlinear transformation, thereby generating predicted muscle strength values. In the training phase of the network, each training sample includes a group of input data and corresponding target output. while calculating the error between the network’s predicted output and the actual target output. The error is calculated as the difference between the network’s predicted output and the actual target output. To reduce this error, the algorithm updates the weights and thresholds between input and hidden layers, as well as between hidden and output layers, using gradient descent. After multiple rounds of training, when the prediction error decreases to the lowest point, the corresponding network parameters are decided. The training process ends accordingly. The trained neural network is able to process similar input samples and output nonlinear transformation results that minimize errors. The output of each layer of neurons is shown in Equation (6).

(6) $$o_{j}^{\left(l\right)}=f^{\left(l\right)}\left(\underset{i}{\sum }w_{ij}^{\left(l\right)}o_{i}^{\left(l-1\right)}+b_{j}^{\left(l\right)}\right)$$

In Equation (6), $o_{j}^{\left(l\right)}$ represents the output of the l-layer neuron j. $f^{\left(l\right)}$ represents the activation function. $w_{ij}^{\left(l\right)}$ represents the weight. $b_{j}^{\left(l\right)}$ represents the bias. The error between the predicted and the actual outputs is represented as a loss function, as displayed in Equation (7).

(7) $$L=\frac{1}{n}\sum_{i=1}^{n}(y_{i}-{\hat{y}}_{i}{)}^{2}$$

In Equation (7), n signifies the sample size. $y_{i}$ signifies the target output. ${\hat{y}}_{i}$ signifies the predicted output. The gradient calculation of the loss function for each weight is shown in Equation (8).

(8) $$\frac{\partial L}{\partial w_{ij}^{\left(l\right)}}=\frac{\partial L}{\partial o_{j}^{\left(l\right)}}\cdot \frac{\partial o_{j}^{\left(l\right)}}{\partial z_{j}^{\left(l\right)}}\cdot \frac{\partial z_{j}^{\left(l\right)}}{\partial w_{ij}^{\left(l\right)}}$$

In Equation (8), $z_{j}^{\left(l\right)}$ represents the input of the l-layer neuron. Gradient descent finds the minimum objective function by taking the derivative of the objective function [29]. Compared with large-scale numerical matrices, gradient descent follows a more efficient iterative solution. For some cases where the least squares method cannot calculate the globally unique optimal solution, gradient descent can still effectively search for the minimum point [30]. Therefore, the study updates the weights and biases based on gradient descent, as shown in Equation (9).

(9) $$\left\{\begin{array}{l} {w^{*}}_{ij}^{\left(l\right)}=w_{ij}^{\left(l\right)}-\eta \frac{\partial L}{\partial w_{ij}^{\left(l\right)}}\\ {b^{*}}_{j}^{\left(l\right)}=b_{j}^{\left(l\right)}-\eta \frac{\partial L}{\partial b_{j}^{\left(l\right)}} \end{array}\right\}$$

**Figure 5:**
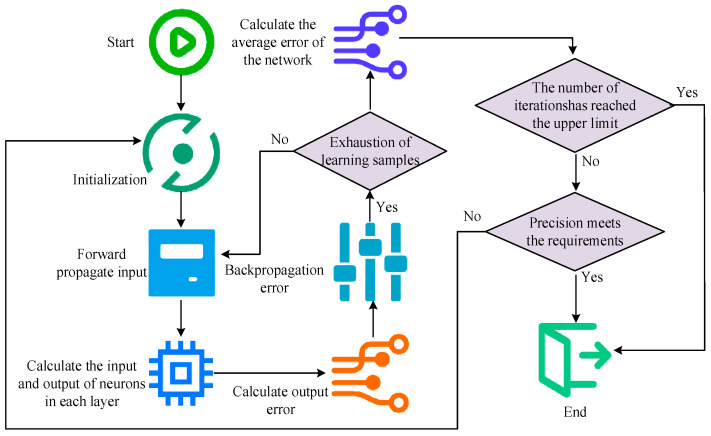
Training steps of BP.

In Equation (9), η represents the learning rate. ${w^{*}}_{ij}^{\left(l\right)}$ represents the updated weight. ${b^{*}}_{j}^{\left(l\right)}$ represents the updated bias. The BP training process mainly involves two stages: forward propagation of signals and backward propagation of errors. Firstly, in the former, input samples are passed from the input layer, processed by each hidden layer, and then passed to the output layer. If the actual output does not match the expected output, it enters error propagation. Error BP is the process of backpropagating the output error in some form to the input layer through a hidden layer, and distributing the error to all units to obtain the error signal. This error signal serves as the foundation for correcting the unit weight. The training steps of BP are shown in Figure 5.

In Figure 5, first, the network parameters, such as weights and biases, are initialized. Secondly, the network output is calculated through forward propagation, and the loss function is applied to evaluate the error between the output and the actual target. The error is propagated back to the network through BP to calculate the gradient for each parameter. Based on these gradients, the weights and biases are updated in the direction of reducing errors. Finally, this process is repeated until the network meets the accuracy requirements on the training data or the maximum iteration is obtained, and the training is complete. It is crucial to choose a suitable muscle strength prediction and evaluation model to ensure that the predicted results are as close to the actual situation as possible. The Pearson correlation coefficient (PCC) is easy to calculate. Therefore, it is applied to measure the correlation between two variables, ranging from −1 to 1, where 1 signifies complete positive correlation, 0 signifies no correlation, and −1 indicates complete negative correlation. The covariance between X and Y is shown in Equation (10).

(10) $$\mathrm{Cov}(X,Y)=\frac{\sum_{i=1}^{n}\left(x_{i}-\bar{x})(y_{i}-\bar{y}\right)}{n-1}$$

In Equation (10), $x_{i}$ and $y_{i}$ represent the observed values between samples X and Y. $\bar{x}$ and $\bar{y}$ represent the mean values between samples X and Y. $Cov\left(X,Y\right)$ signifies the covariance between samples X and Y. The standard deviation between samples X and Y is shown in Equation (11).

(11) $$\left\{\begin{array}{c} \sigma (X)=\sqrt{\frac{\sum_{i=1}^{n}(x_{i}-\bar{x}{)}^{2}}{n-1}}\\ \sigma (Y)=\sqrt{\frac{\sum_{i=1}^{n}(y_{i}-\bar{y}{)}^{2}}{n-1}} \end{array}\right\}$$

In Equation (11), $\sigma \left(X\right)$ and $\sigma \left(Y\right)$ signify the standard deviations between samples X and Y. The correlation between muscle strength and actual strength can be represented by PCC r, as calculated in Equation (12).

(12) $$r=\frac{\mathrm{Cov}(X,Y)}{\sigma (X)\sigma (Y)}$$

**Figure 6:**
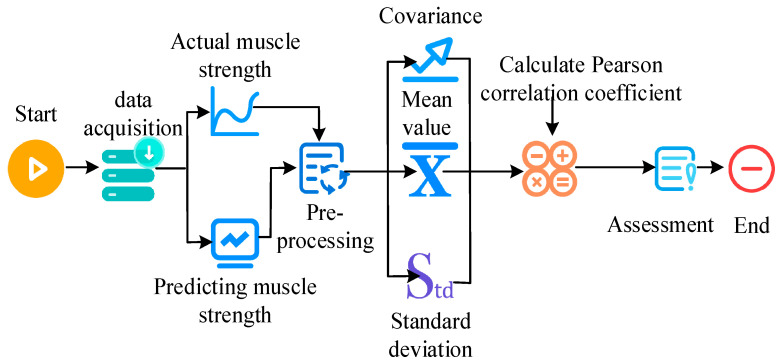
Steps for evaluating muscle strength prediction based on Pearson correlation coefficient.

When two variables are directly proportional, the PCC is positive. When they are inversely proportional, the PCC is negative. When there is no linear relationship, the correlation coefficient approaches zero. The steps for evaluating muscle strength prediction based on PCC are as follows. First, actual muscle strength measurements and corresponding predicted values are collected and preprocessed. Next, the mean, covariance, and standard deviation of actual and predicted muscle strength are collected. Relying on the calculated covariance and standard deviation, the PCC between actual muscle strength and predicted muscle strength is obtained. Finally, the performance of the muscle strength prediction model based on the PCC is evaluated. The steps for evaluating muscle strength prediction based on PCC are shown in Figure 6.

### 2.3. Experimental Parameter Settings

**Figure 7:**
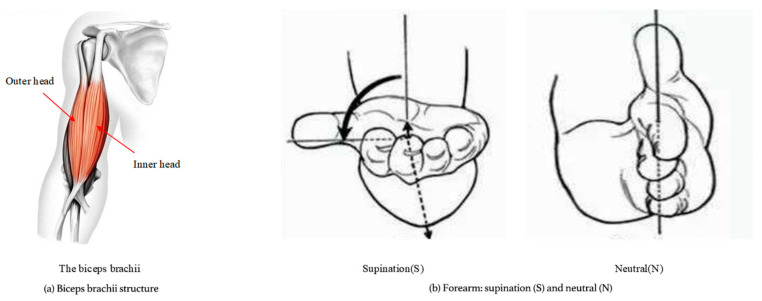
Biceps and elbow flexion posture of the arm.

To validate the effectiveness of the proposed ICA-BP for locating key MTUs and predicting muscle strength during human motion, this study recruited 10 healthy male participants aged between 23 and 25 years, with an average height of 175.6 cm and a weight range of 64–68 kg. All participants avoided vigorous physical activity within 24 h prior to the experiment. The study protocol was reviewed and approved by the Institutional Review Board of Qingdao University (Protocol No. QDU-IRB-2023-145). All procedures were conducted in accordance with the Declaration of Helsinki, and all participants provided written informed consent prior to participation. The biceps brachii was selected as the target muscle in this study for two main reasons: (1) it serves as the primary driving muscle for upper limb elbow flexion, with well-defined functional anatomy; (2) it is located superficially, making it convenient for sEMG electrode placement, and the resulting signals exhibit high quality, which is suitable for muscle force modeling. During the test sessions, participants were instructed to perform standard elbow flexion movements in a seated position. Two forearm postures were examined: the neutral position (N position) and the supine position (S position). For each posture, participants completed five full cycles of elbow flexion, each lasting approximately 3 s, with a 5 s rest between repetitions to prevent fatigue. As illustrated in Figure 7, different postures result in distinct activation patterns of the medial and lateral MTUs within the biceps brachii. The medial MTU is more active in the S position, while the lateral MTU dominates in the N position. sEMG signals were recorded throughout the movement process and normalized using Maximal Voluntary Contraction (MVC) tasks to ensure signal comparability and accurate muscle force estimation.

For sEMG signal acquisition, disposable Ag/AgCl surface electrodes (10 mm diameter) were placed using a bipolar configuration with a 20 mm inter-electrode distance, following SENIAM guidelines. Skin areas were cleaned with 70% alcohol and shaved if necessary to reduce impedance. To minimize motion artifacts, electrodes were secured with medical adhesive tape, and subjects performed elbow flexion tasks while maintaining a stable seated posture. The sEMG signals were normalized using the Maximal Voluntary Contraction (MVC) method. Each participant performed three MVC trials for the biceps brachii, each lasting approximately 5 s, with a 2 min rest interval between trials to avoid fatigue. The highest value obtained across the three trials was used as the normalization reference for subsequent sEMG amplitude calculations.

The experiment was conducted on a Windows 10 platform with an Intel Core i9-13900K processor, 16 GB DDR4 RAM, and an NVIDIA RTX 4080 GPU (16 GB VRAM). MATLAB R2020a was used as the software environment. The research divided the collected data into a training set and a test set at an 8:2 ratio, with 80% of the data used for model training and the remaining 20% used for model testing. Furthermore, to enhance the robustness of the results, five-fold cross-validation was adopted in the training phase in this paper. The average error index was calculated in each round of training, and the final performance was summarized on the test set. The experimental parameter settings are summarized in Table 1.

## 3. Results

### 3.1. Effects of Key MTU Localization in Human Motion Based on ICA Algorithm

**Figure 8:**
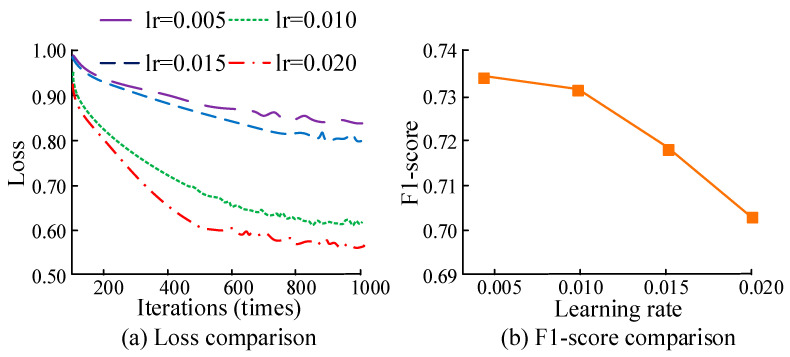
Comparison of loss value and F1-score of ICA algorithm under different learning rates.

The learning rate is crucial for the performance of the ICA algorithm, as it determines the magnitude of weight adjustment during the optimization process. Therefore, this study set the learning rate to different values. The loss value F1-score was used for evaluation to obtain the optimal learning rate, as presented in Figure 8. From Figure 8a, when the iteration reached 700 times, the loss curves of different learning rates tended to be stable. When the learning rate was 0.020, the loss value was the smallest. At 0.020, the loss value decreased with the increase in the learning rate. In Figure 8b, the F1-score value decreased as the learning rate increased. At 0.020, the F1-score value was optimal.

**Figure 9:**
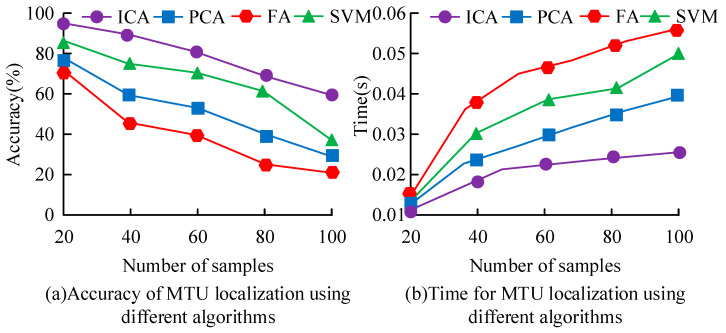
Comparison of accuracy and time for MTU localization using different algorithms.

Under different sample sizes, the accuracy and time of locating the key MTUs of human motion based on ICA were compared with other algorithms, as presented in Figure 9. From Figure 9a, the accuracy of different algorithms in locating the key MTUs of human motion decreased continuously with increases in sample size. The accuracy of locating the key MTUs of human motion based on ICA was the highest, while the accuracy of locating the key MTUs of human motion based on Factor Analysis (FA) was the lowest. The reason for this is that FA usually requires a large sample size to generate reliable results and performs poorly for tasks with less data. When the sample size was 20, the accuracy rates were 98% and 71%, respectively. In Figure 9b, the accuracy of different algorithms in locating the key MTUs of human motion increased continuously with increases in sample size. The ICA showed good performance in locating the key MTUs of human motion, with the shortest localization time. When the sample size was 100, the localization time was 0.025 s.

The error of locating the key MTUs based on the ICA algorithm was compared with the error of locating the key MTUs using other algorithms, as displayed in Table 2. In Table 2, the performance of locating the key MTUs of human motion based on the ICA algorithm was the best, with the smallest errors in both N and S postures. When the sample size was 20, the error was minimized, with errors of 1.24% and 2.33%, respectively. The performance of locating the key MTUs based on FA was the worst, because FA is very sensitive to initial values and optimization algorithms. Different initial values or algorithms may lead to different results, thereby increasing errors. When the sample size was 100, the errors were 3.87% and 3.67%, respectively.

### 3.2. The Effect of Muscle Strength Prediction and Evaluation of the Key MTUs in Human Motion

**Figure 10:**
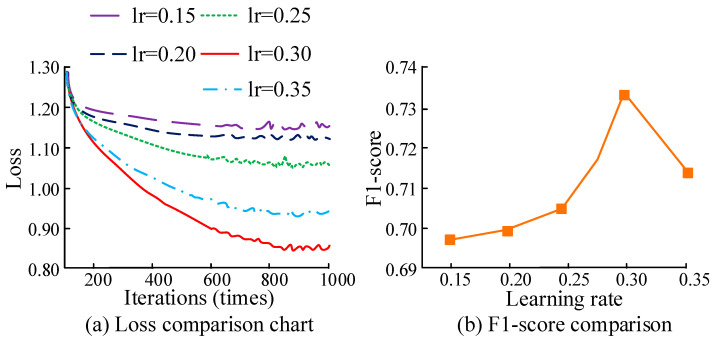
Comparison of loss values and F1-scores of BP neural networks at different learning rates.

The size of the learning rate directly affects the convergence speed and state of the BP. Excessive learning rate may cause the model to oscillate near the optimal solution and fail to converge. A low learning rate may lead to a slow convergence speed, and may even cause the model to become stuck in local optimal solutions. Therefore, to obtain the optimal learning rate, this study set the learning rate to different values and evaluated it through the loss value and F1, as displayed in Figure 10. From Figure 10a, when the iteration reached 800 times, the loss curves of different learning rates stabilized. The loss value was minimized when the learning rate was 0.3. The loss value declined when the learning rate was below 0.3. When the learning rate was greater than 0.3, the loss value increased. In Figure 10b, when the learning rate was 0.3, the F1-score was optimal. When the learning rate was below 0.3, the F1-score increased with the learning rate. When the learning rate exceeded 0.3, the F1 decreased.

The recall rate and F1-score value of muscle strength prediction based on the BP neural network for key MTUs were compared with those of other algorithms, as displayed in Table 3. As shown in Table 3, the BP showed good performance in predicting muscle strength for key MTUs, with the highest recall rate and F1-score value, and the smallest error, which were 88%, 0.90, and 2.34%, respectively. The reason is that the BP neural network is trained through the BP algorithm, which can effectively adjust the network weights to reduce prediction errors. The performance of muscle strength prediction based on the Random Forest (RF) algorithm for key MTUs of human motion was poor, with the lowest recall and F1-score values, and the largest errors of 75%, 0.80, and 3.98%, respectively. The reason is that RF is affected by the inherent model complexity and feature selection limitations when processing nonlinear and high-dimensional data, resulting in lower recall rates and F1-score values.

**Figure 11:**
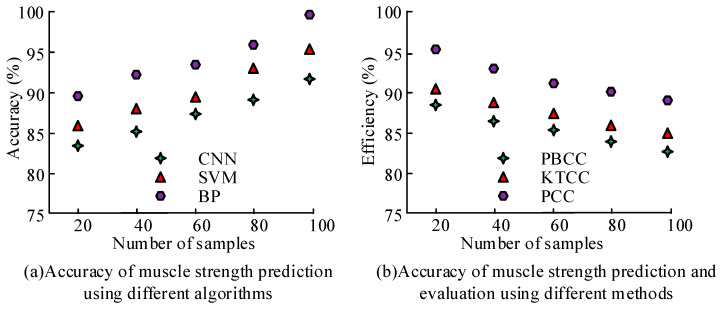
The accuracy and efficiency of muscle strength prediction for key MTUs of human motion using different algorithms.

The accuracy and efficiency of the designed method for predicting muscle strength based on key MTUs of human motion were compared with other methods, as displayed in Figure 11. As shown in Figure 11a, the accuracy of different algorithms in predicting muscle strength based on key MTUs of human motion increased continuously with the increase in sample size. The accuracy of muscle strength prediction based on BP for key MTUs was the highest, while the accuracy of muscle strength prediction on the basis of a Convolutional Neural Network (CNN) for key MTUs of human motion was the lowest. The reason is that CNN usually requires rich training data to learn effective feature representations and performs poorly on tasks with limited data. When the sample size was 100, the accuracy rates were 99% and 91%, respectively. As shown in Figure 11b, the efficiency of different methods in evaluating key MTUs of human motion for muscle strength prediction decreased continuously with the increase in sample size. The efficiency of evaluating key MTUs for muscle strength prediction based on PCC was the highest, while the efficiency of evaluating key MTUs based on Point-Biserial Correlation Coefficient (PBCC) was the lowest. The reason for this is that when there are missing values in the dataset, the PBCC calculation is affected, and additional processing steps are required to handle the missing data. When the sample size was 20, the efficiency was 96% and 88%, respectively.

**Figure 12:**
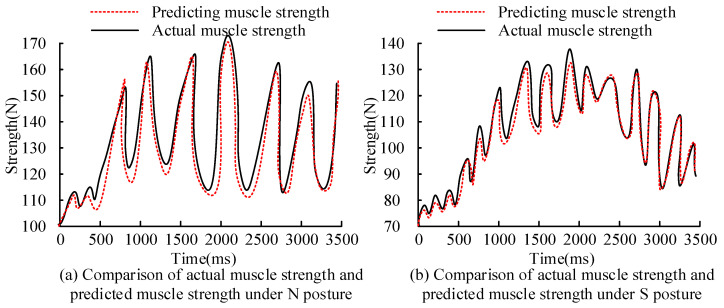
Comparison of actual muscle strength and predicted muscle strength under different postures.

The comparison between actual muscle strength and predicted muscle strength under different postures is displayed in Figure 12. In Figure 12a, in the N posture, the predicted muscle strength curve for predicting key MTUs of human motion is basically consistent with the actual muscle strength curve. At 900 ms, the error between the predicted muscle strength curve and the actual muscle strength was the largest. The actual muscle strength was 123 N, the predicted muscle strength was 116 N, and the error was 5.7%. In Figure 12b, in the S posture, the predicted muscle strength curve is basically consistent with the actual muscle strength curve. At 1800 ms, the error between the predicted muscle strength curve and the actual muscle strength was the largest. At this time, the actual muscle strength was 137 N, the predicted muscle strength was 132 N, and the error was 3.6%. The error of muscle strength prediction based on BP for key MTUs of human motion in the N posture and S posture was less than 6%, which verifies the effectiveness of the proposed method.

## 4. Discussion

The muscle strength prediction method that integrated ICA-based key MTU localization with a BP neural network model demonstrates strong performance in localizing key MTUs and predicting muscle strength. Experimental results show that the proposed method maintained high accuracy and stability across different postures and sample sizes. In the localization task, ICA achieved a localization error of only 1.24% when the sample size was 20, significantly outperforming traditional dimensionality reduction methods, such as PCA and FA. This superior performance can be attributed to the fact that ICA is able to separate independent components from high-dimensional mixed sEMG signals, thereby extracting target muscle activities more accurately and avoiding misidentification caused by overlapping or redundant signals. In contrast, PCA can only ensure that components are uncorrelated but cannot guarantee true statistical independence, making it difficult to effectively separate different muscle activities when dealing with non-Gaussian and non-stationary sEMG signals; FA, on the other hand, relies on a large sample size and is highly sensitive to initial values and optimization processes, often resulting in instability under small-sample and high-noise conditions. Therefore, ICA demonstrates stronger adaptability and robustness in the separation of complex physiological signals, which leads to lower localization errors in this study.

In addition, the muscle strength prediction error in this study was 5.7%, which falls within the acceptable error range in rehabilitation medicine and sports training. It is generally recognized that when the standard error of measurement (SEM%) is below 10%, the measurement error is considered small and clinically acceptable. For example, Morin et al. reported SEM% values ranging from 0.50% to 3.45% in a hand-held dynamometry-based muscle strength assessment study, indicating that errors of this magnitude are entirely within the acceptable range [31]. This precision is sufficient to provide a reference for rehabilitation training, guiding patients to gradually increase the load and avoid over-training. It is also applicable to real-time feedback systems in sports training, used to monitor muscle strength output, optimize training movements, and prevent sports injuries. Therefore, the proposed model demonstrates promising potential for practical application in rehabilitation and sports training contexts. However, since the method has not yet been clinically validated, its real-world applicability requires further investigation.

Compared with the hybrid musculoskeletal modeling method proposed in [15], the method in this study does not rely on complex anatomical reconstruction tools, such as OpenSim, quickly predicting muscle strength without requiring detailed physiological modeling. This gives it clear advantages in terms of generalizability and computational efficiency. Unlike the machine learning strategy in [16], which depends heavily on extensive feature engineering, the BP neural network model used in this study can directly learn the mapping relationship from raw sEMG signals in an end-to-end fashion, effectively reducing human-induced errors. Moreover, while the non-parametric functional muscle network proposed in [17] has value in fatigue monitoring, it focuses more on state tracking and trend analysis, and is less suitable for real-time muscle strength prediction. In contrast, the ICA-BP integrated model proposed in this study demonstrated superior predictive performance, achieving a prediction accuracy of 99% with a sample size of 100, a maximum error of only 5.7%, a recall of 88%, an F1-score of 0.90, and maintaining millisecond-level response speed, thereby reflecting higher prediction accuracy and real-time responsiveness.

Nevertheless, several limitations of this study should be acknowledged. First, experimental data were collected from only 10 healthy male participants, and the experiments focused solely on a single movement involving the biceps brachii (elbow flexion), which limits the generalizability of the findings. Future studies should expand the sample size to include subjects of different genders, ages, and muscle health conditions to verify the broader applicability. Second, the input data used in this study were limited to unimodal surface EMG signals, without considering multisource data fusion. Given the pronounced spatiotemporal complexity of muscle activity, future research may draw on the multimodal approach of Hwang et al. [32]. In their study, surface EMG signals were combined with inertial measurement unit (IMU) data and processed using a CNN-LSTM-Attention model for fatigue detection, achieving 87.94% classification accuracy and 87.94% balanced recall across 35 participants, thereby significantly improving robustness in personalized monitoring. This suggests that integrating additional sensors, such as IMUs or mechanomyography (MMG), could support multidimensional modeling of motor intent and muscle function. In addition, the proposed method has only been validated in an offline simulation environment and has not yet been deployed in actual rehabilitation devices or prosthetic systems, lacking systematic evaluation of response time, latency, and operational stability.

## 5. Conclusions

The results demonstrate that the proposed model achieved high prediction accuracy under both neutral (N) and supinated (S) arm positions, with average errors of 1.24% and 2.33%, respectively, and a maximum error not exceeding 5.7%. Compared with traditional methods, the proposed ICA-BP neural network model can more accurately identify the most actively engaged muscle–tendon units during movement and simultaneously improve predictive performance. Future work will focus on reducing the computational complexity of ICA and validating the model under diverse postures and muscle groups to enhance its applicability in rehabilitation assessment and intelligent training systems.

## Figures and Tables

**Table 1:** Parameter settings for key MTU localization and muscle strength prediction in human motion.

MTU Localization		Muscle Strength Prediction	
Parameter	Value	Parameter	Value
Sampling rate	1000 Hz	Hidden layers	2
Filter settings	20–450 Hz	Number of neurons per layer	5
Window size	0.5 s	Training set size	80%
Step size	0.2 s	Test set size	20%
Number of components	5	Target error	0.001
Number of iterations	1000	Momentum	0.9
Convergence threshold	0.0001	Max epochs	1000

**Table 2:** Errors of MTU localization by different algorithms under different postures.

Number of Samples	Positioning Error Under N Posture				Positioning Error Under S Posture			
	ICA	PCA	FA	SVM	ICA	PCA	FA	SVM
20	1.24%	2.98%	1.99%	1.64%	2.33%	3.45%	2.89%	2.59%
40	1.46%	3.12%	2.18%	1.87%	2.56%	3.67%	3.01%	2.73%
60	1.78%	3.44%	2.45%	2.03%	2.79%	3.89%	3.22%	2.95%
80	2.63%	2.76%	3.06%	2.58%	3.06%	4.05%	3.50%	3.31%
100	2.55%	3.58%	3.87%	3.12%	3.42%	4.24%	3.67%	3.56%

**Table 3:** Recall rate and F1-score of muscle strength prediction using different algorithms.

Pooling Strategy	Recall (%)	F1-Score	Error (%)
BP	0.88	0.90	2.34
SVM	0.80	0.85	2.79
RF	0.75	0.80	3.98
XGBoost	0.85	0.92	2.45
LSTM	0.82	0.88	2.56
CNN	0.79	0.83	3.37

## Data Availability

Dataset available on request from the authors.
